# Thermokinetic and Chemorheology of the Geopolymerization of an Alumina-Rich Alkaline-Activated Metakaolin in Isothermal and Dynamic Thermal Scans

**DOI:** 10.3390/polym16020211

**Published:** 2024-01-11

**Authors:** Raffaella Aversa, Laura Ricciotti, Valeria Perrotta, Antonio Apicella

**Affiliations:** Advanced Materials Laboratory, Department of Architecture and Industrial Design, University of Campania Luigi Vanvitelli, Via San Lorenzo, 81031 Aversa, Italy; laura.ricciotti@unicampania.it (L.R.); valeria.perrotta@unicampania.it (V.P.)

**Keywords:** chemorheology, geopolymer, viscoelasticity, DSC thermograms deconvolution, additive manufacturing

## Abstract

Alkaline sodium hydroxide/sodium silicate-activating high-purity metakaolin geopolymerization is described in terms of metakaolin deconstruction in tetrahedral hydrate silicate [O[Si(OH)_3_]]^−^ and aluminate [Al(OH)_4_]^−^ ionic precursors followed by their reassembling in linear and branched sialates monomers that randomly copolymerize into an irregular crosslinked aluminosilicate network. The novelty of the approach resides in the concurrent thermo-calorimetric (differential scanning calorimetry, DSC) and rheological (dynamic mechanical analysis, DMA) characterizations of the liquid slurry during the transformation into a gel and a structural glassy solid. Tests were run either in temperature scan (1 °C/min) or isothermal (20 °C, 30 °C, 40 °C) cure conditions. A Gaussian functions deconvolution method has been applied to the DSC multi-peak thermograms to separate the kinetic contributions of the oligomer’s concurrent reactions. DSC thermograms of all tested materials are well-fitted by a combination of three overlapping Gaussian curves that are associated with the initial linear low-molecular-weight (Mw) oligomers (P1) formation, oligomers branching into alumina-rich and silica-rich gels (P2), and inter- and intra-molecular crosslinking (P3). The loss factor has been used to define viscoelastic behavioral zones for each DMA rheo-thermogram operated in the same DSC thermal conditions. Macromolecular evolution and viscoelastic properties have been obtained by pairing the deconvoluted DSC thermograms with the viscoelastic behavioral zones of the DMA rheo-thermograms. Two main chemorheological behaviors have been identified relative to pre- and post-gelation separation of the viscoelastic liquid from the viscoelastic solid. Each comprises three behavioral zones, accounting for the concurrently occurring linear and branching oligomerization, aluminate-rich and silica-rich gel nucleations, crosslinking, and vitrification. A “rubbery plateau” in the loss factor path, observed for all the testing conditions, identifies a large behavioral transition zone dividing the incipient gelling liquid slurry from the material hard setting and vitrification.

## 1. Introduction

The growing interest in understanding the science and technology relationships behind geopolymerization chemo-physical properties [1,2,3] is related to the potential use of geopolymers as greener materials for greener production technologies [4,5,6,7].

Concrete is the most widely used material in construction due to its excellent mechanical performance, durability, and low cost [5,6]. However, its use raises worldwide concerns associated with the high consumption of natural resources in cement manufacturing [6,7] and a high amount of CO_2_, accounting for 5–7% of CO_2_ anthropogenic global emissions and 3% of the global greenhouse effect [8,9]. Therefore, less energy harvesting and polluting characteristics of geopolymer production can satisfy the urgent need for cement substitutes to reduce environmental concerns [9,10,11]. Moreover, new green technologies, such as additive manufacturing, can be used with these thermosetting polymeric ceramics [4]. Due to its potential high productivity and reduced environmental and production costs [4,12], additive technology is becoming increasingly popular in different areas [4,10], including its applications in the construction industry [8,9,10,11,12,13] and, recently, a biomimetic and sustainable approach to developing biomedical applications [13,14].

The most widely used additive manufacturing technique in the construction sector is extrusion-based 3D printing, in which the object is built by depositing material extruded layer-by-layer from a moving nozzle [4,12]. The knowledge of the reacting geopolymeric slurry viscoelasticity evolution during the processing is a critical parameter to control in extrusion-based additive manufacturing [4,5,12]. The proposed chemorheological approach relating the chemical evolution and the associated changes of the slurry viscoelastic characteristics could help define the correct 3D-printing process parameters. As occurs for thermosetting organic polymeric composites, a geopolymeric reactive ceramic extrusion needs chemical and rheological evolution knowledge during all processing phases [15]. Viscosity, gelation time, and mechanical properties may vary with the reacting slurry composition, temperature, flow condition, and deposition rate, according to the kinetics of the chemical reactions occurring in the geopolymer-activated paste [16,17].

From the chemical perspective, alkaline metal salts and hydroxides (sodium silicate and NaOH) are needed to dissolve the metastable aluminosilicate and catalyze the polycondensation reaction [17,18,19]. During the alkaline-activated dissolution [16,17], the chemical bonds of the kaolin metastable aluminosilicate are fragmented down into hydrate silicate [OSi(OH)_3_]^−^ and aluminate [Al(OH)_4_]^−^ ionic precursors [3,16] starting the geopolymerization process by a recombination of these dissolved units [19,20].

**Figure 1:**
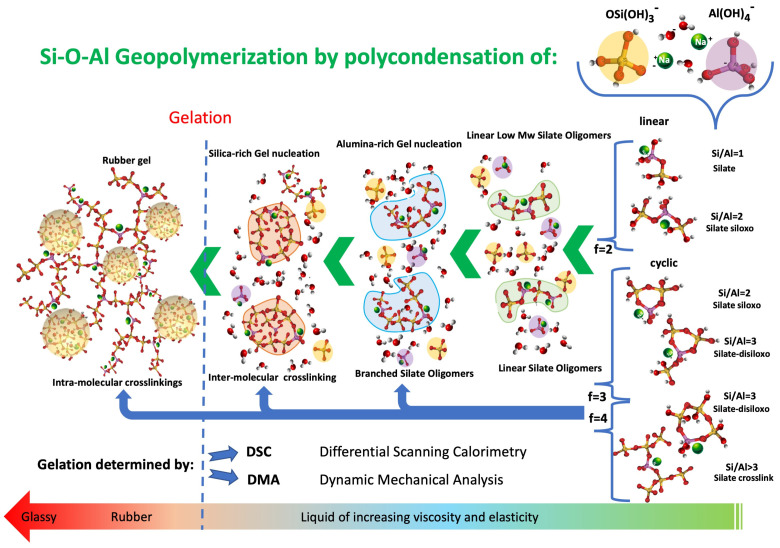
Mechanism of geopolymerization: sketch of the reaction paths and the reacting medium physical modifications.

Figure 1 sketches the phases of an alkaline-activated metakaolin geopolymerization from its initial liquid to final glassy states (from right to left sides in Figure 1).

In particular, the tetrahedral hydrate silicate [O[Si(OH)_3_]]^−^ and [Al(OH)_4_]^−^ ionic precursors (reported in the upper right side of Figure 1) reassemble themselves in linear and branched macromolecules [2,3], generating the initial inorganic monomeric molecules [3,18]. However, the relatively slow dissolution rate of metakaolin compared to the formation rate of the oligomeric species, which are characterized by rates of the order of milliseconds [4,19], is the parameter controlling the sialate oligomers actual availability rate. Both dissolution and low Mw oligomer formation occur in the very early stages, and they could be comprised in the first oligomeric species formation.

The concurrent presence of activated silica and alumina units is mandatory for geopolymerization reactions to occur [16] since, although Al^3+^ as a modifier ion cannot independently participate in the formation of a polymeric network, it can replace Si^4+^ in the [SiO_4_]^4−^ tetrahedron when presenting four-oxygen coordination [20,21,22]. In the forming polymeric network, the excess negative charge of Al in tetrahedron monomeric ions [AlO_4_]^5−^ (compared to the [SiO_4_]^4−^) is balanced by a positive alkali ion such as Na^+^ and K^+^ [4,18].

Using ^29^Si and ^27^Al NMR spectroscopy, North and Swaddle [2] confirmed Davidovits’ [1] hypothesis of the presence of solute species with Si–O–Al sialate linkages in concentrated solutions. The kinetics of monomeric acyclic sialate species formation were reported to be more rapid than those of the cyclic one. Moreover, the oligo-sialate polymerization, taking place on a time scale of around 100 milliseconds, was 100 to 1000 times faster than the ortho-silicate polymerization.

Copolymerization of the sialate, sialate–siloxo, and sialate–disiloxo monomers generated by the dissolution of metakaolin was then favored over the polymerization of ordered silicate structures, impairing their ability to form crystalline domains [2,3].

Exothermic polymerization reactions develop, and then macromolecular structures consist of disorderly interlinked alumina and silica tetrahedra sharing oxygen atoms that are concurrently present in the reacting paste [16,17,23].

In the early stages, sialates (Si/Al = 1), sialate–siloxo (Si/Al = 2), and sialate–disiloxo (Si/Al = 3) linear or cycle monomers are generated. At the same time, oligomer crosslinks are formed when Si/Al = or >3 (reported on the right side of Figure 1) [3]. Kinetics investigation of silicate exchange in alkaline aluminosilicate solutions [2] reported the presence of one sialate monomer, two sialate–siloxo monomeric species (one linear with a functionality two and a second cyclic of functionality three), and two cyclic sialate–disiloxo monomeric units of functionalities three and four able to promote, as occurring for organic polymers, branching and crosslinking [24,25,26].

Such monomers connect to create oligomers of progressively higher Mw as the metakaolin dissolution and polymerization by condensation reactions progress [27,28]. Sodium cations (indicated as green spheres in Figure 1) continue to be incorporated in the growing macro-molecule to keep the structure neutral. Due to the presence of longer and branched macromolecules, the progressive process of macromolecular growth is accompanied by a corresponding increase in the reacting liquid slurry viscosity and elasticity.

As depicted in the central portion of Figure 1, sialate, sialate–siloxo, and sialate–di-siloxo molecules form linear polymer chains when behaving as difunctional (f = 2) until the Si/Al ratio is lower than two. However, when the Si/Al ratio is two or higher, cyclic sialate branching and macromolecular crosslinks are generated (lower right side of Figure 1), leading to the formation of macromolecules with functionalities higher than four (f > 4) oligomers [1,2,3,22,23] that rapidly increases the branching tendency of the growing macromolecule [26].

From the thermodynamic side of the neo-forming inorganic polymeric solution, the initially low Mw oligomeric chains are progressively hindered as polycondensation proceeds due to the modification of the solution’s dynamic (rheology) [22] and thermodynamic (solubility) properties [24]. The progressively weaker interaction of the solvating units with the growing macromolecules reduces their affinity with the solvent and increases their tendency to segregate from the solution. These adverse thermodynamic conditions favor the nucleation of “aluminate-rich gel” particles containing the first formed alumina-rich linear sialate [28,29,30,31,32,33]. As the polycondensation reaction continues, [Al(OH)_4_]^−^ concentration in the reacting medium reduces, increasing the Si/Al ratio above three, further favoring molecular branching and precipitation of Si-rich gel particles [27,28]. The alumina- and silica-rich gel particles then aggregate into larger particulate structures that drastically affect the material’s rheological [22] and ultimate properties [29,30,31].

The irregular aluminosilicate backbone generated by the random copolymerization of linear and branched sialate, sialate–siloxo, and sialate–disiloxo avoids the early crystallization process while favoring the formation of an amorphous crosslinked rubber network [32,33,34]. The point where this event occurs marks the end of a liquid-like behavior and announces the transition into a solid elastic rubber identified in Figure 1 as “Gelation”. The still-containing reactive oligomers rubber network continues to react, increasing the crosslinking density and finally allowing geopolymer vitrification [22].

The steps mentioned above could occur in parallel with the reaction mechanism of the alkali-activated materials. Their relative weight on the final structure is statistically dependent on the temperature of polymerization [34,35].

According to the previously described complexity of the concurrent chemical and rheological phenomena, mathematical modelling and complete chemorheological characterizations become necessary if we want to optimize the conditions of a 3D printing process where extrusion and deposition are ended before gelation occurs while the material should be able to self-sustain once deposited [4,22,35].

The relationship between process and material relaxation time must be accounted for when analyzing the feasibility of an extrusion process using a material characterized by a specific viscoelastic behavior. This is usually achieved by considering a dimensionless number, the Deborah number (De), which compares the time the material takes to flow or respond to applied stresses or deformations and the time scale of the experiment or process [36,37]. If a rheological process is faster than the relaxation process, the material will appear elastic; otherwise, the viscous part will dominate. Measurements in the elastic region (high De number) provide information about the material’s internal structure, e.g., molecular or morphological. In contrast, when the viscous region (low De number) is considered, meaningful information about the flow behavior for correct material processing will be acquired [4,22].

Using organic or inorganic thermosetting materials that undergo chemical structure modifications during their processing, the ongoing chemical and morphological changes and the corresponding proportion and relative influence on material viscous and elastic response could be expected to impair the analysis using the De number. An alternative experimental technique of continuously monitoring the viscoelastic materials’ mechanical response time delays to imposed deformations or loads has been applied. Namely, we intend to develop the bases for the process control and assess its feasibility under specific flow or applied stress conditions by analyzing the ratio between the viscous and elastic material response using DMA oscillatory shear experiments [22]. Due to the intrinsic characteristics of this test, we will be able to exploit the results in terms of viscous (loss) and elastic (storage) components derived from the experimentally measured complex viscosities and shear moduli according to the mechanical response time delay (δ) of a reacting slurry to an imposed oscillatory deformation.

The innovative aspects of the proposed research reside in the modelling of the chemorheological behavior of these inorganic polymers obtained by pairing deconvolution of differential scanning calorimetry (DSC) thermograms to identify the main chemical events, and the corresponding viscoelastic response defined in dynamic mechanical analysis (DMA); both run at the same temperature conditions.

## 2. Materials and Methods

### 2.1. Materials and Sample Preparation Procedures

High-purity metakaolin (ASTM C-618 [38] class N pozzolans) (MetaMax^®^ BASF, New York, NY, USA) and sodium silicate solution (Prochin Italia S.r.l, Caserta, Italy) are used to prepare the alkali-activated reacting slurry. The composition of the raw metakaolin and sodium silicate solution is reported in Table 1. The alkaline activating solution has been prepared by dissolving reagent-grade anhydrous sodium hydroxide pellets (Sigma-Aldrich, St. Louis, MO, USA) in the sodium silicate solution according to the procedures described in previous papers [22,39].

The weight ratio of sodium hydroxide and sodium silicate in the activating solution was 1/7.7. The solution was equilibrated at room temperature for 24 h before use [22,39]. Metakaolin powder was added to the alkaline-activating solution in a liquid-to-solid ratio of 1/1.7 and then mechanically mixed and sonicated for 10 min before testing. Cold mixing at 5 °C was adopted to avoid geopolymerization reactions during preparation [2,3,22]. The mixing procedures were conducted at 5 °C using a thermostat bath.

The composition of the resulting geopolymeric system, determined by energy dispersive spectroscopy (EDS) in previous research [39], is Al_2_O_3_ 3.48 SiO_2_ 1.0 Na_2_O 12.14 H_2_O with a molar ratio silica/alumina of 3.48.

Geopolymer composition table, EDS, and X-ray characterizations of the raw materials and resulting geopolymer have been determined in a previous paper [39] and reported in the Supplementary Data Document attached to this paper.

### 2.2. Thermoanalysis Methods and Testing Procedures

The freshly made paste was simultaneously tested using the differential scanning calorimeter (DSC) and the dynamical mechanical analyzer (DMA). The DSC and DMA equipment were controlled by Mettler STAR^e^ Software V. 18.0 using a multichannel input interface for parallel data exchange. A dynamic heating rate of 1 °C/min and isothermal cure temperatures of 20 °C, 30 °C, and 40 °C were utilized for mechanical and calorimetric characterization.

#### 2.2.1. Differential Scanning Calorimetry

The heat released during the geopolymerization of thermally scanned and isothermally cured samples has been monitored by a Mettler ADSC differential scanning calorimeter equipped with an intra-cooler unit driven by the Mettler software STAR^e^ V 18.0.

Dynamic temperature scans were run from 5 °C to 95 °C at a heating rate of 1 °C/min. Isothermal tests were conducted at 20 °C, 30 °C, and 40 °C. In temperature scan tests, about 30 mg of alkaline-activated metakaolin freshly prepared pastes were poured and sealed into medium-pressure crucibles (Mettler MP 120 μL, max pressures 2 MPa) to avoid water evaporation during the test and then readily placed in the DSC oven for testing. The isothermal tests were carried out using aluminum crucibles. All tests were performed under a purging nitrogen gas flow of 14 mL/min. Steel and aluminum crucible weights and internal surfaces were controlled for corrosion after preliminary tests; no evidence of corrosion was noted.

The advancement of the geopolymeric reaction has been expressed as a fractional value of the final heat of the reaction (evaluated from the total area under thermograms) and the measured partial area at a determined temperature or time during the thermal scan.

All thermal scans were replicated on six freshly made samples. Statistical analysis was performed on each set of derived data to define their means and standard deviations.

The one-way ANOVA has been considered to compare the means of the total heat released in the different polymerization conditions to determine whether there was statistical evidence that the associated population means were significantly different. In the analysis of variance, the null hypothesis, indicating that the group means are equal, was tested using the F test. A significant level of *p* ≤ 0.05 was adopted to reject the null hypothesis.

#### 2.2.2. DSC Thermograms Deconvolution

A phenomenological approach described by Bampi et al. has been used to extrapolate more information from the DSC thermograms [40]. The contributions of concurrent geopolymerization reactions have been separated considering the DSC traces to be composed of the sum of Gaussian functions:

(1) $$\sum y_{i}=\sum a_{i}{exp}^{-\frac{{\left(x-bi\right)}^{2}}{{2c}^{i2}}}+di$$

where:

ai, is the peak amplitude (in mW);
bi, is the peak temperature or time (in °C for dynamic and hours for isotherm tests);
ci, is the peak with (in °C for dynamic and hr for isotherm tests);
di, is the peak ordinate baseline (in mW);
x, is the thermogram abscissa (in °C for dynamic or hr, for isothermal tests);
y, is the thermogram ordinate (in mW).

The tabular format of the row data of the DSC thermograms is first imported into the Excel spreadsheet. The Gaussian functions (Equation (1)) were generated in the same spreadsheet following the time sequence reported in the DSC row data containing about 45,000 intervals. A least squares methodology was used to make a numerical fit using the sum of single Gaussian functions to overlap the experimental DSC thermogram as closely as possible. The values of the partial functions and the respective sum are updated together with the differences between the experimental and calculated values of the respective squared deviations by manually adjusting the values of “peak temperature/time”, “width”, and “amplitude” of each peak. The process is repeated until no change in the sum of the squared deviations evaluated by the Excel Solver tool regression analysis is reached. The deconvolution of the peaks and the estimates of the temperatures and enthalpies of each single overlapping peak have been performed for the thermograms of the dynamic temperature scans and isothermal tests.

#### 2.2.3. Dynamic Mechanical Analysis Test Procedures

The dynamic-mechanical analyses have been run on a Mettler Toledo dynamic mechanical analyzer (DMA-SDTA 1+) operating in shear force (max 0.5 N) and displacement (max 10 μm) control modes and at a frequency of 10 Hz.

Complex, storage and loss viscosities, shear moduli, and loss tangents of the alkali-activated metakaolin paste during the temperature scans from 25 to 95 °C and isothermal 24 h cure at 20 °C, 30 °C, and 40 °C were continuously monitored from its initial liquid to its final solid state.

**Figure 2:**
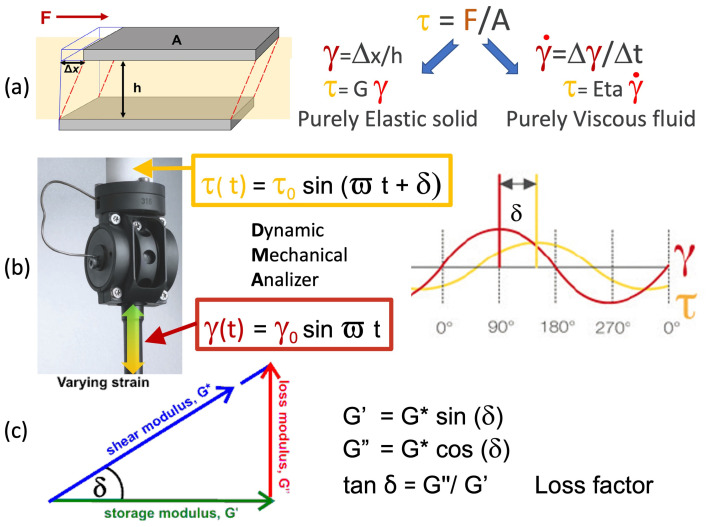
Viscoelasticity principles and analysis tools: (**a**) shear behaviors in purely viscous liquid and purely elastic solid; (**b**) DMA shear test tool and viscoelastic behavior measurements; (**c**) vector representation of complex shear modulus and storage and loss components relationships.

Figure 2 shows the DMA set-up and viscoelastic analysis principles. Two identical weight paste specimens are symmetrically sandwiched in the shear sample assembly with a 1.0 to 1.5 mm gap between three circular steel plates of 10 mm diameter. The two stationary sample assembly outer parts are connected with a force sensor. At the same time, the central one moves at a sinusoidal strain of controlled amplitude and an oscillation frequency of 10Hz (pulse ω = 2π f = 62.8 rad/s).

The results of our modulated shear tests may be equivalently represented using shear dynamic viscosities (Eta″, Eta′, and Eta″ in Pa*s) as well as shear moduli (G″, G′, and G″ in MPa) according to

Eta′ = G″/ω Eta″ = G′/ω(2)

Moreover, data can also be analyzed by referring to the ratio between the loss and storage mechanical components designated as loss factor = tanδ. This parameter provides a measure of the relative predominance of the dissipative (tanδ > 1) and elastic (0 < tanδ < 1) character of the material:

tanδ = G″/G′ = Eta′/Eta″(3)

Each thermo-mechanical analysis was replicated on five new freshly made samples. Statistical analyses were performed on each set of derived data to define their means, standard deviations, and significant differences.

## 3. Results

### 3.1. Differential Scanning Calorimetry

DSC temperature scans and isothermal cure tests monitored the heat evolution from freshly made alkaline-activated metakaolin mixes.

#### 3.1.1. DSC Temperature Scan Test

**Figure 3:**
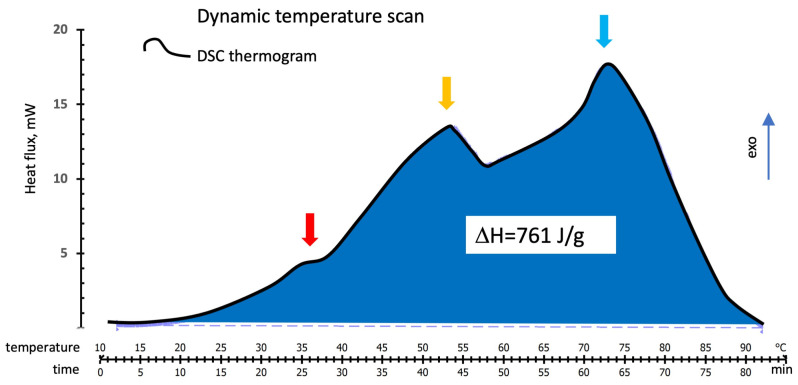
DSC temperature scan on the alkaline-activated metakaolin reactive mix. Red, yellow, and blue arrows indicate the presence of shoulder and peaks in the thermogram. The “exo” arrow indicates the direction of exotherms.

A typical thermogram of the heat released during the temperature scans at 1 °C/min is shown in the thermogram in Figure 3. The thermogram presents shoulders or peaks identifying three zones (the red, yellow, and blue arrows at about 37 °C, 53°C, and 73 °C, respectively), suggesting the incidence of different concurrent thermal events.

These thermal events can be associated with the linear and branching oligomerization, and crosslinking reactions depicted in Figure 1.

The tested samples’ mean overall heat of the reaction calculated from the area under the thermograms is 761 ± 21 J/g. Heat flux starts at about 20 °C and ends just above 90 °C. A second temperature scan on the same cured sample heated to 120 °C showed no additional residual reactivity.

#### 3.1.2. DSC Isothermal Cure Tests

**Figure 4:**
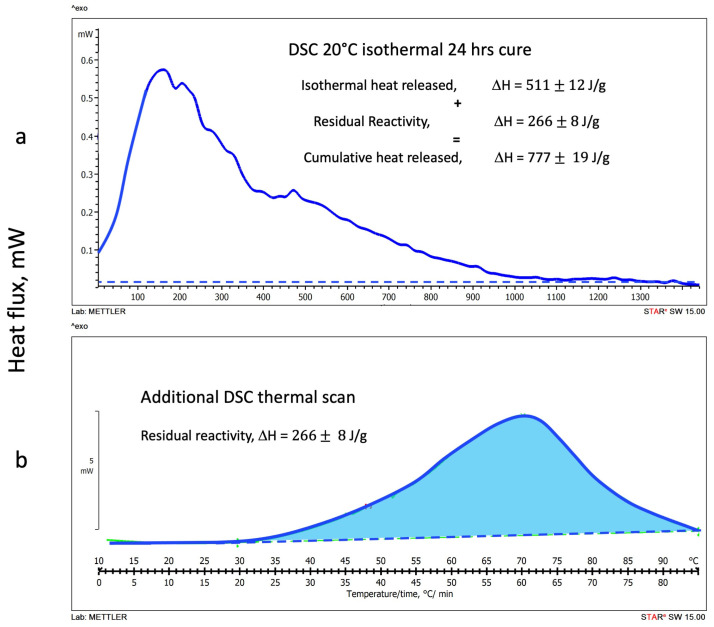
Alkaline activated metakaolin geopolymerization (**a**) DSC 20 °C Isothermal cure thermogram (**b**) Second temperature scan to evaluate the residual reactivity. The term “exo” indicates the direction of the exotherms.

**Figure 5:**
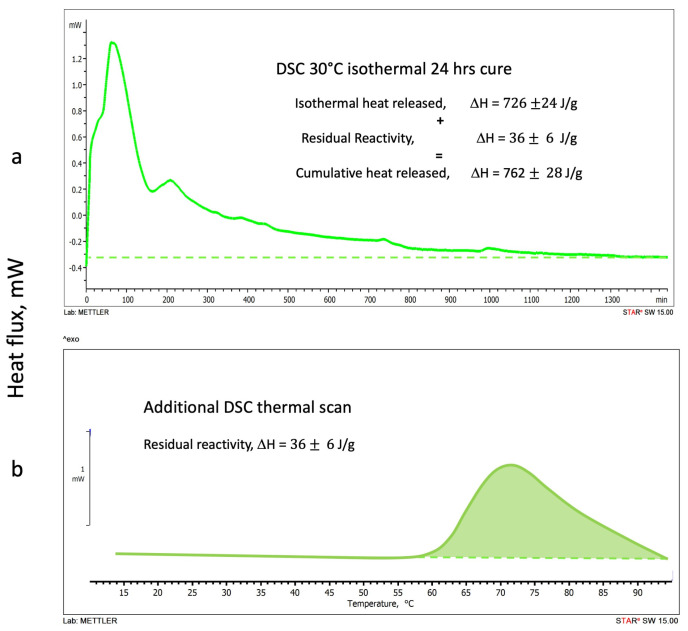
Alkaline-activated metakaolin geopolymerization: (**a**) DSC 30 °C isothermal cure thermogram, (**b**) second temperature scan to evaluate the residual reactivity. The term “exo” indicates the direction of the exotherms.

**Figure 6:**
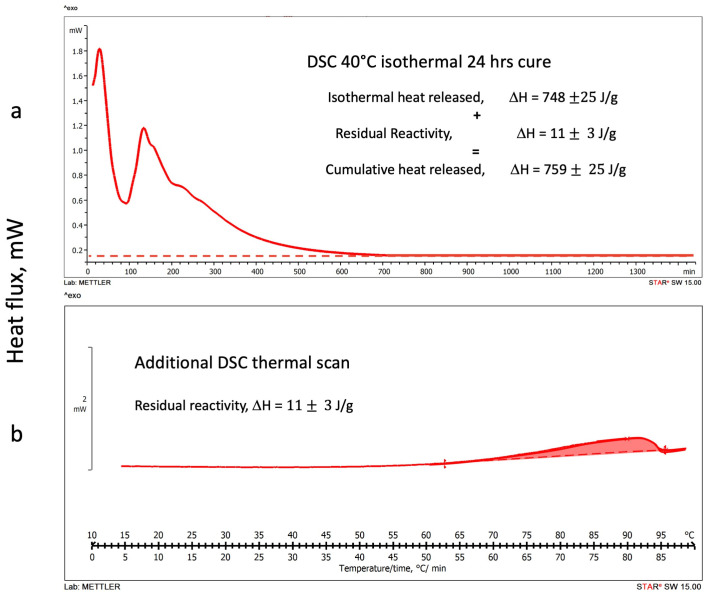
Alkaline-activated metakaolin geopolymerization: (**a**) DSC 40 °C isothermal cure thermogram, (**b**) second temperature scan to evaluate the residual reactivity. The term “exo” indicates the direction of the exotherms.

Thermograms of the heat released during the DSC isothermal scans at 20 °C, 30 °C, and 40 °C are shown in Figure 4a, Figure 5a and Figure 6a, respectively. All isothermal tests were carried out for 24 h. No significant heat fluxes were detected after this cure time, indicating that the geopolymer setting reactions did not proceed further after this period. Although geopolymers have been described to further modify their chemical and mechanical properties for up to 30 days [41,42], the chemical or physical modifications that occur in the long-term geopolymer setting have not been considered in this study, which is principally aimed to investigate the rheological and chemical behavior in the very early stages of the polymerization [42].

The residual reactivities of each isothermally cured geopolymeric sample were examined by running a second DSC temperature scan at 1 °C/min. Figure 4b, Figure 5b and Figure 6b report the thermograms of the second scans on the same figure of the corresponding isothermal test.

A significant residual reactivity heat is released by the sample cured at 20 °C (Figure 4b). Namely, the heat released during the isothermal cure is 511 J/g, while about 266 J/g were released during the second scan. According to the thermogram trace initial inflexion, reactions restart above 25 °C. The partial heat released in the isothermal and dynamic scans and the cumulative amount, ΔH = 777 J/g, are reported in Figure 4b.

Figure 5 shows (a) the 30 °C isothermal cure and (b) the second temperature scan thermograms. The heat released in the isothermal cure is 726 J/g while the residual one is 36 J/g, significantly lower than the 20 °C cure. The cumulative heat of the reaction is ΔH = 762 J/g.

Finally, Figure 6 shows the thermograms of (a) the 40 °C isothermal cure and (b) the second temperature scan. The heat released in the isothermal cure is 748 J/g, while the residual one is only 11 J/g with a final cumulative ΔH = 759 J/g.

The thermogram traces reported in Figure 4, Figure 5 and Figure 6, as detailed in Figure 3 for the DSC temperature dynamic scans, present evident shoulders and peaks that may be associated with concurrent reaction.

Table 2 summarizes the heat released (ΔH total) in the different polymerization conditions.

The one-way analysis of variance at *p* < 0.05 for the experimental and the model mean values of the heat released from the dynamic and isothermal tests have been applied. No significant differences existed between the mean total heat released values for the experimental DSC tests run in the temperature scan and isothermal polymerizations. The same table also compares the values of the partial and total reaction heats evaluated from the Gaussian functions model that are described in the next section.

### 3.2. DSC Thermograms Deconvolution

Reaction degrees and kinetics of the reactants in alkaline-activated geopolymerization closely correlate to their rheological behavior during setting, final mechanical properties, and durability [21,22]. However, quantifying the reactions using conventional methods is challenging, primarily when different reaction mechanisms are concurrently occurring (as described in Figure 1), and the thermo-calorimetric traces present shoulders and peaks.

**Figure 7:**
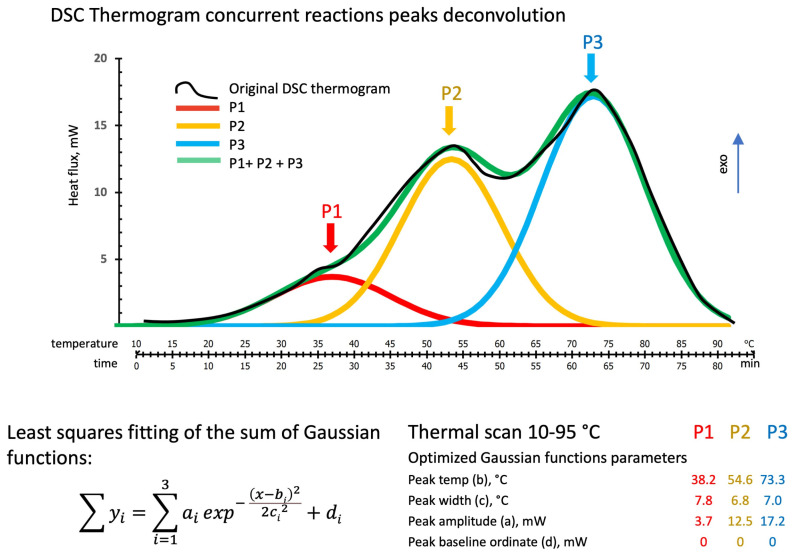
Peaks deconvolution of the DSC thermogram for a dynamic temperature scan: least square fitting of the sum of Gaussian functions by peaks (P1, P2, and P3) amplitude (a), temperature (b), with (c), and baseline (d) parameters adjustments. The term “exo” indicates the direction of the exotherms.

**Figure 8:**
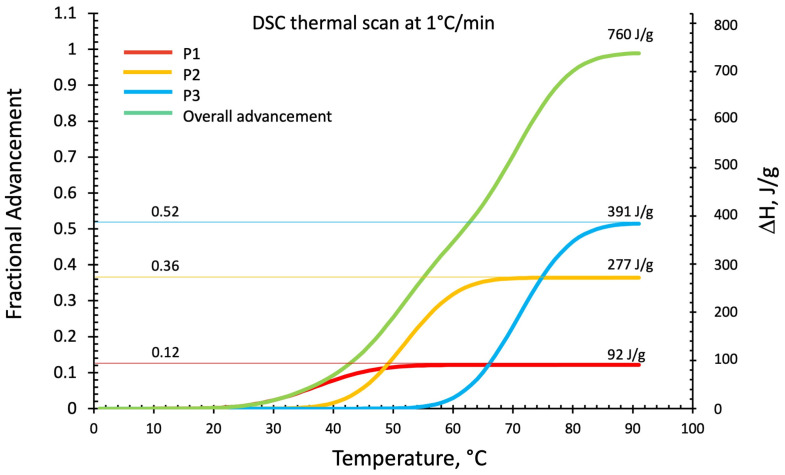
Fractional advancement and final reaction enthalpies (ΔH) for each deconvoluted Gaussian peak and their sum.

**Figure 9:**
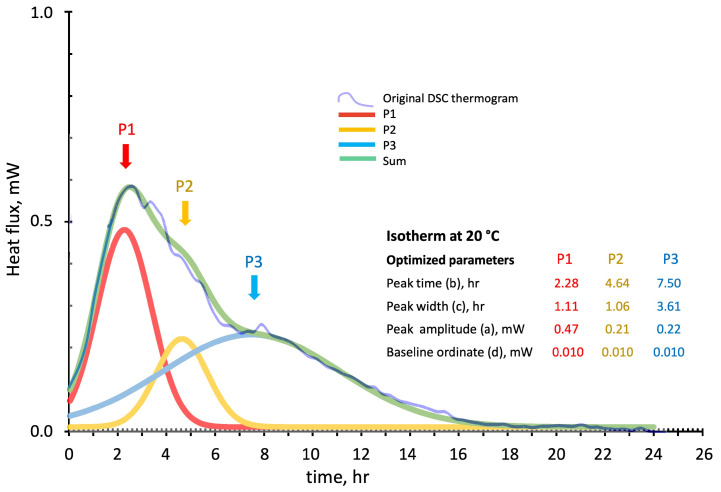
Peak deconvolution of the 20 °C isothermal cure DSC thermogram: least square fitting of the sum of three Gaussian functions by peaks (P1, P2, and P3) (a) amplitude in mW, (b) time in hour, (c) width in hour, and (d) baseline in mW parameters adjustments.

**Figure 10:**
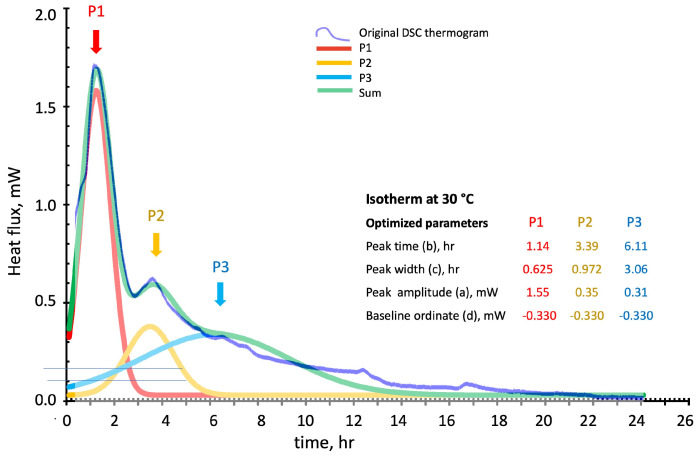
Peak deconvolution of the 30 °C isothermal cure DSC thermogram: least square fitting of the sum of three Gaussian functions by peaks (P1, P2, and P3) (a) amplitude in mW, (b) time in hour, (c) with in hour, and (d) baseline position in mW, parameters adjustments.

**Figure 11:**
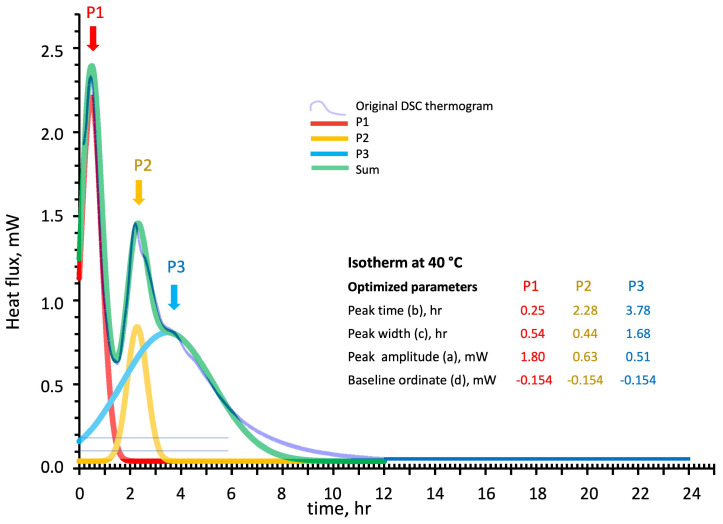
Peak deconvolution of the 40 °C isothermal cure DSC thermogram: least square fitting of the sum of three Gaussian functions by peaks (P1, P2, and P3) (a) amplitude in mW, (b) time in hour, (c) with in hour, and (d) baseline in mW, parameters adjustments.

**Figure 12:**
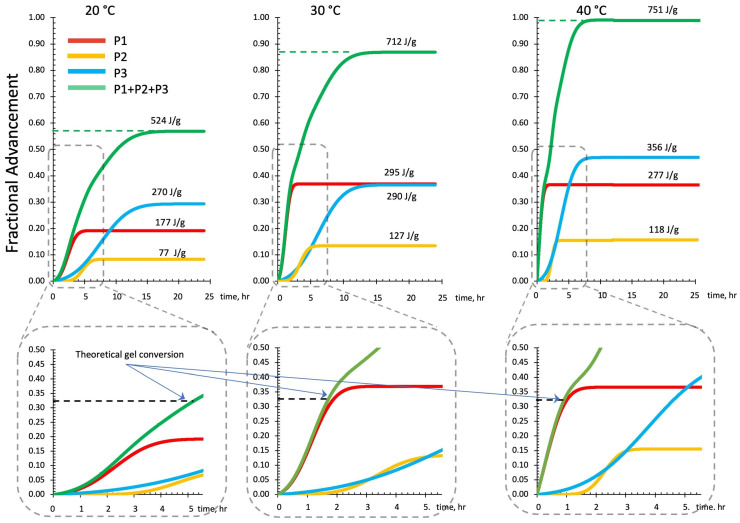
Fractional advancements and final reaction enthalpies (ΔH) from each deconvoluted Gaussian peak and their sum from the DSC thermograms of isothermal cure (20 °C, 30 °C, 40 °C). Curve detail (lower part of Figure 12) indicates the time needed to reach the theoretical gel point (critical conversion α_c_ = 0.333) for each cure temperature.

The proposed peak deconvolution method using the sum of three Gaussian functions has been applied to both temperature scans and isothermal tests. Results are shown in Figure 7, Figure 8, Figure 9, Figure 10, Figure 11 and Figure 12.

#### 3.2.1. DSC Temperature Scan Test

Figure 7 shows the deconvolution method applied to the DSC thermogram of a 1 °C/min temperature scan. The black line is the original DSC thermogram, while the individual optimized Gaussian peaks and their sum are indicated in red (P1), in yellow (P2), in blue (P3), and their sum in green. Gaussian functions have been optimized by considering the peak position expressed in °C and peak amplitude and baseline position expressed in mW. The relative contributions and advancements over the overall theoretical (Gaussian functions sum) heat released (normalized to the sample weight and expressed in J/g) have been evaluated for each single peak by its integration over the test time (the test has been carried out at 1 °C/min) and are reported in Figure 8.

The theoretically calculated heat released in our dynamic and isothermal tests are compared in Table 2 with the DSC experimental values.

The agreement between the sum of the three Gaussian functions model and the experimental thermogram of the temperature scan of Figure 6 is good, with a max local discrepancy of 0.9 mW. The experimental to model total heats released are 760 ± 25 vs. 761±27 J/g, respectively.

The relative enthalpic contributions and advancements of the single Gaussian function and their sum are reported in Figure 8. The theoretical enthalpies in J/g have been evaluated for each single peak by integration over the test time (the time/temperature equivalence is reported on the Figure 7 diagram abscissae).

In these dynamic temperature conditions (heating at 1 °C/min) and according to the reaction paths indicated in Figure 1, it can be inferred that the first peak (P1 in red) could be associated with the tetrahedral hydrate silicate [O[Si(OH)_3_]]^−^ and [Al(OH)_4_]^−^ ionic precursors reassembling in linear molecules followed by the formation of low-Mw linear oligomers [34,35]. The second peak (P2 in yellow) could represent the first formation of alumina-rich gels deriving from the branching of the linear oligomers due to the presence of trifunctional and tetrafunctional sialates monomers (reported in the lower right portion of Figure 1). In contrast, the third peak (P3 in blue) may be associated with the same sialates inducing crosslinking in silica-rich gel-growing particles and their further cross-linking in a fully gelled network. This is consistent with Kriven’s study using transmission electron microscopy (TEM) of the microstructure of fully reacted potassium-poly(sialate–siloxo)-type geopolymers [41]. They observed a structure formed by nanoparticles of dimensions ranging from 5 to 15 nm separated by a nanoporosity of 3 to 10 nm. The TEM/EDS analyses of these fully reacted particulate domains show that the most frequently occurring Si/Al ratio is two, corresponding to a polymeric network formed by poly(sialate–siloxo).

Figure 8 reports the fractional advancements of the three hypothesized reaction mechanisms. In these dynamic heating conditions (1 °C/min), the formation of linear oligomers represents only 12% of the overall heat released. At the same time, the branching and alumina-rich gel is 36%, and the sialate–siloxo crosslinking (silica-rich gel) is 52%.

#### 3.2.2. DSC Isothermal Scan Test

Figure 9, Figure 10 and Figure 11 show the deconvolution method applied to the isothermal DSC thermograms run at 20 °C, 30 °C, and 40 °C as a function of the test time expressed in hours. The blue line is the original DSC thermogram, while the individual optimized Gaussian peaks and their sum are indicated as P1 (in red), P2 (in yellow), P3 (in blue), and sum (in green). The Gaussian functions in the isothermal tests have been optimized by considering “Peak position” and “width” expressed in hour and “Peak amplitude” and “baseline position” expressed in mW.

The Gaussian functions models follow the experimental isothermal thermograms of Figure 9, Figure 10 and Figure 11 with maximum local discrepancies of 0.02 mW at 20 °C, 0.07 mW at 30 °C, and 0.09 mW at 40 °C. Discrepancies are more evident at long test times where additional physical events should probably be accounted for, especially for the higher isothermal cure temperatures. Although the disordered structure of the developing silico-aluminate geopolymer favors the formation of a glassy state, nucleation of zeolites in geopolymers has been mentioned in many works. Partial crystallization may be expected for longer, especially when experiencing relatively higher temperatures and, in our case, at high silica contents of the reacting mix [43,44,45].

The theoretical model released heats, calculated from integrating the “Sum” curves, are compared in Table 2, with the data experimentally determined by DSC. Namely, 524 ± 28 J/g vs. the experimental 511 ± 32 J/g at 20 °C, 712 ± 15 J/g vs. the experimental 726 ± 24 J/g at 30 °C, and 751 ± 17 J/g vs. the experimental 748 ± 25 J/g at 40 °C.

The relative enthalpic contributions and advancements of the single Gaussian function and their sum are reported in Figure 12. The theoretical enthalpies in J/g have been evaluated for each single peak by its integration over the test time.

The enthalpic contribution of the third peak (P3 blue line), associated with sialate oligomers branching and the formation of silica-rich gel, progressively increases as the temperature is raised. In contrast, the second peak (P2 in yellow) reaches an apparent stationary relatively low value of the fractional advancement at 30 °C isothermal cure. The first peak (P1 in red), associated with the initial recombination reaction and formation of linear oligomers, presents a significant increase on moving from 20 °C to 30 °C.

#### 3.2.3. DSC Kinetics from Deconvoluted Peaks

Curing temperature is a relevant factor in the setting of the geopolymer structure and properties [45]. Reaction mechanisms of geopolymers are differently activated by the temperature while co-occurring [46]. To determine the temperature dependence of these three reactions associated with the Gaussian functions, the variations of the three peaks’ maxima positions (the time needed to reach the peak maximum t_max_) have been taken as a measure of the half-time of the reaction and the kinetic evaluated as k_1/2_ = 1/t_max_ at each test temperatures and treated using the Arrhenius approach for thermally activated processes:

k_1/2_ = Ae^−Ea/RT^, (4)

where:

- k_1/2_ is the rate constant;
- T is the absolute temperature (in °K);
- A is a constant reaction frequency factor;
- E_a_ is the activation energy for the reaction (in J mol^−1^);
- R is the universal gas constant, 8.314 J mol^−1^ °K^−1^.

**Figure 13:**
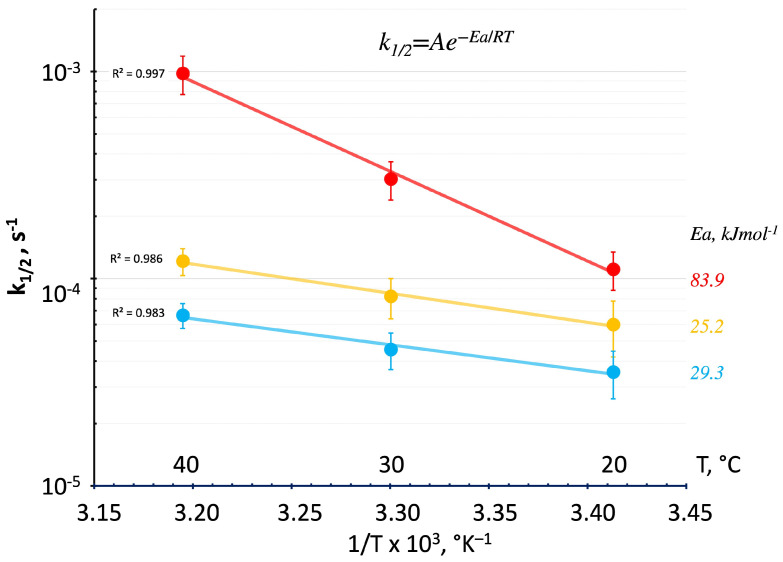
Arrhenius plot of the half-time reaction kinetics evaluated for the three Gaussian functions that describe isothermal geopolymerization of the alkaline-activated metakaolin: red line P1, yellow line P2, and blue line P3. Activation energies and linear regression coefficients of determination R^2^ are also reported on the curves.

Apparent activation energies as a function of estimated reaction advancement have been described in the literature to vary from initially high values and then decrease to a lower stationary value as the degree of reaction increases [22]. Such a behavior can be justified by the concurrent reactions identified by the contributions of the single Gaussian functions reported in Figure 13, where the kinetic constants are plotted in a semilogarithmic scale as a function of the inverse of the absolute temperature. The half-time reaction kinetics were taken from the optimized “peak time” parameters relative to P1, P2, and P3, which were evaluated for the 20 °C, 30 °C, and 40 °C isothermal geopolymerization reported in Figure 9, Figure 10 and Figure 11. The slope of the linear regressions for the three groups of data reported in Figure 13 as red, yellow, and blue lines for P1, P2, and P3, respectively, indicates activation energy relative to the early reactions (linear oligomerization P1) of 83.9 kJmol^−1^, and significantly lower values of the activation energies result for P2 and P3 (which correspond to the formation of alumina rich and silica gels), namely, 25.2 kJmol^−1^ and 29.3 kJmol^−1^, respectively.

Although chemical control is the basic assumption of all statistical treatments of the cure reactions, on some occasions, the cure may also be controlled by physical factors such as diffusion constraints or dissolution rate (as is the case of the alkaline attack on the metastable metakaolin layered structure) [42]. The alkaline attack starts on the outer faces of the metakaolin particles continuing from the edges to the inside.

The study by North [2] used ^29^Si and ^27^Al NMR spectroscopy, which clarified the existence of solute species with Si–O–Al sialate linkages in the alkaline-activated metakaolin solutions, with oligo-sialate polymerization taking place on a time scale of about 100 milliseconds. The reaction is so fast at room temperature that metakaolin dissolution becomes the rate-determining factor of the sialate oligomer formation in the early polymerization stages (corresponding to our observed first Gaussian peak).

Moreover, the diffusion constraints in the liquid slurry of the progressively larger molecules deriving from the polycondensation of sialate oligomers are affected more strongly than those of small molecules. This diffusive constraint reduces reaction rates depending on the type of molecules involved in those reactions. Due to the steric hindrance, the potentially reactive sites become less available for the reaction being shielded by the surrounding network topology to grow in complexity. The diffusion constraints become more relevant in the crosslinked rubber and, further on, during the geopolymer vitrification stages. The average reaction rates may decrease by orders of magnitude due to diffusion limitation due to the decrease in the free volume caused by the formation of the polymer network. This is the case of the reactions of linear oligomerization in the early stage of polymerization associated with the first Gaussian peak versus those of the branched molecules related to the second and third peaks of deconvoluted DSC thermograms.

This diffusion constraint mechanism justifies the higher half-time reaction kinetics activation energy observed for the first reaction peak (i.e., 83.9 kJ mol^−1^) versus those observed for the second and third peaks (25.2 and 29.3 kJmol^−1^, respectively) that are consistent with diffusion-controlled processes in rubber to glassy polymers [47].

#### 3.2.4. Residual Reactivity from DSC Temperature Scan Test of Isothermally Cured Samples

**Figure 14:**
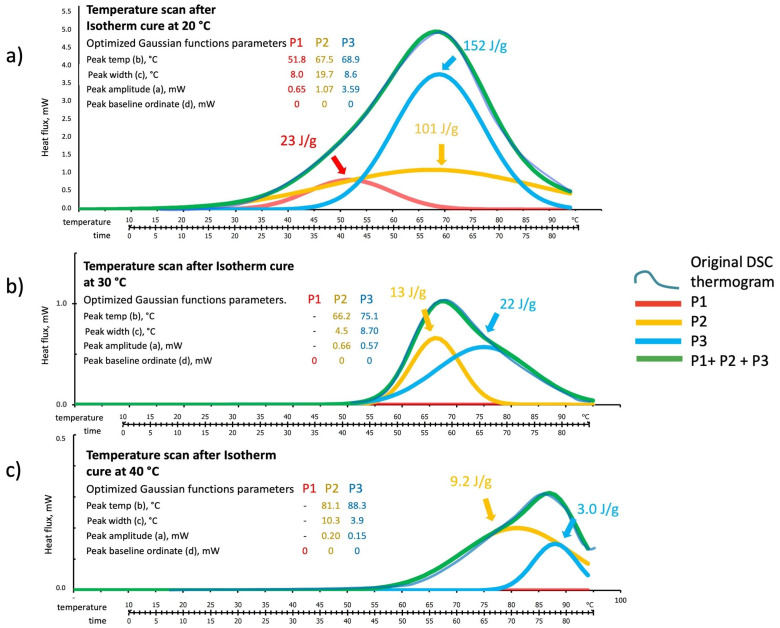
Residual reactivity after isothermal cure. Peaks deconvolution of the DSC thermograms of temperature scans on samples previously isothermally cured at (**a**) 20 °C, (**b**) 30 °C, and (**c**) 40 °C.

To investigate the kinetics and chemical nature of the residual reactivity of the isothermally cured samples, the deconvolution method has been applied to analyze the DSC thermograms of the temperature scans of Figure 9b, Figure 10b and Figure 11b. Results for the residual reactivities of the three isothermal cures are shown in Figure 14.

The original thermogram traces of the residual reactivities (identified in Figure 14 as dark blue lines) are also well-fitted by the Gaussian functions sum model in all three temperatures investigated. The relative enthalpic contribution of each single Gaussian peak is indicated on the curves.

The residual reactivity thermogram of the sample cured for 24 h at 20 °C is closely described by the presence of all three hypothesized Gaussian functions, while the first peak (P1) is not present for the samples cured at 30 °C and 40 °C.

It can be argued from this observation that the 20 °C cure does not exhaust the reactions associated with the formation of low-Mw linear oligomers (see Figure 1) [22], leaving a still-active ability to form linear oligomers if a post-cure at higher temperatures is experienced. Conversely, in the polymerizations at 30 °C and 40 °C, the linear oligomerization reaction associated with the Gaussian peak 1 (red curves in Figure 7, Figure 8, Figure 9, Figure 10, Figure 11, Figure 12 and Figure 13), and according to its higher activation energy, readily overcomes the branching reaction related to the formation of the alumina and silica-rich gels.

According to the values of the heat released by the isothermal cure and residual reactivity scans, the partial and final fractional conversions for each cure temperature profile are reported in Table 3.

Transforming the fractional conversion data of Table 3 in the percentage of the total heat released, we analyzed the relative weight of each of the three peaks in the different polymerization thermal conditions investigated.

In the dynamic temperature scan (1 °C/min), only 12% can be associated with forming the linear oligomer, while branching reactions into alumina-rich and silica-rich gels are 36% and 52%.

The isothermal cure at 20 °C followed by the temperature scan to exhaust the still-reactive species, allows 26% of the enthalpy of the reaction associated with linear oligomerization (P1) and 23 and 51% related to the branching of alumina and silica gels.

At 30 °C, the percentage of linear oligomerization (P1) rises, accordingly, its higher activation energy (see Figure 13), to 39%, while the branching reactions (P2 and P3) represent 19% and 42% of the reaction’s contributions.

A similar situation has been observed at 40 °C with 36% due to the linear oligomerization (P1) and 17% and 47% to the branching reactions for alumina-rich (P2) and silica-rich (P3) gel formation.

The different relative balances between linear and branched oligomer formation at varying thermal cure conditions can strongly influence the rheology of the system during early-stage geopolymerization and the properties of the final hard-set material.

Further investigations have been then devoted to analyzing the parallelism between the deconvoluted DSC thermograms and the viscoelastic rheological behavior that has been described in our previous paper to be characterized by six significantly different rheological zones that occur during DMA temperature scan [22].

## 4. Discussion

The DSC and DMA thermograms regarding the occurrence of calorimetric and rheological events have been compared and discussed. The paper aimed to correlate the chemical events occurring in the alkaline-activated metakaolin slurry and the physical events, such as its viscosity, elasticity time dependency, and gelation time, which are critical factors for the correct use of these materials in the additive manufacturing technologies [48,49,50]. The feasibility in ordinary and variable external environmental conditions of geopolymers 3D printing processes [51] is strongly conditioned by the availability of models to predict its processability for variable temperature and composition [52,53,54,55,56] and final properties [57,58,59].

### 4.1. Thermosets Polymerisation and Theoretical Evaluation of the Gelation Critical Conversion

The authors successfully applied the Flory–Stockmayer gelation theory [15,22] to describe the branching and gelation of the step-growth polymerization and incipient gelation of geopolymers and thermosetting organic polymers containing multifunctional molecules using the critical conversion (α_c_) [25,26,54,55]:

α_c_ = 1/(f − 1), (5)

which correlates the functionalities of the molecules of a reactive solution able to form linear polymeric chains (when functionality is two) or branching and crosslink (when functionality is equal or higher than two) [25,26,27,28,29,54,55]. The presence of tetrafunctional molecules, favored in our systems where the value of Si/Al is higher than three [22], provoke macromolecules to branch up to gelation. In our alkaline-activated metakaolin thermosetting mix, the reassembly of the hydrated silicate [O[Si(OH)_3_]]^−^ and aluminate [Al(OH)_4_]^−^ tetrahedral ionic species may lead to the formation of either difunctional (f = 2), trifunctional (f = 3), and tetrafunctional (f = 4) molecules (reported on the right side of Figure 1).

According to Equation (5), the critical conversion α_c_ could be between 0.33 to 0.50 (functionalities four and three, respectively). Until this critical value of the chemical conversion is reached, the reacting mixture behaves as a processable fluid. Beyond this threshold, it cannot flow any longer. However, although not further extrudable in a deposition process, the rubber gel must still exhaust from 50.0 to 77.7% of its reactivity, progressively increasing the intra-molecular crosslinking density up to vitrification into a solid glass.

The critical values range of the conversions is reported in Figure 12 as dotted lines. It indicates the minimum theoretical time span (when f = 4) needed to reach gelation in the different thermal curing conditions.

### 4.2. Parallel Interpretation of Dynamic Mechanical Analysis and Deconvoluted Differential Scanning Calorimetry Thermograms

The parallel analysis of the DSC deconvoluted peaks with thermomechanical tests is aimed at coupling each DSC thermal event with the viscoelastic physical changes detected by the dynamic thermomechanical analysis.

Namely, in our previous study on the evolution of the viscoelastic characteristics of the same reacting geopolymer [22], we identified six behavioral transition zones in the DMA rheo-thermogram:

- Zone I: Kaolin deconstruction and silico-aluminate oligomers formation—viscoelastic liquid;
- Zone II: Nucleation of alumina-rich gel particles—a viscoelastic liquid solution containing alumina-rich gel particles;
- Zone III: Nucleation of silica-rich gel particles—a viscoelastic liquid solution containing alumina-rich and silica-rich gel particles;
- Zone IV: Silico-aluminate rubber gel—amorphous viscoelastic rubber;
- Zone V: Silico-aluminate vitrification starting (still crosslinking can occur);
- Zone VI: Fully polymerized silico-aluminate glassy geopolymer.

The separation of the exothermic contributions obtained by using the Gaussian deconvolution method allows us to associate each behavioral zone with the corresponding reaction mechanism.

#### 4.2.1. Dynamic Mechanical Analysis and Differential Scanning Calorimetry Peaks Deconvolution in Temperature Scans at 1 °C/min Heating Rate

**Figure 15:**
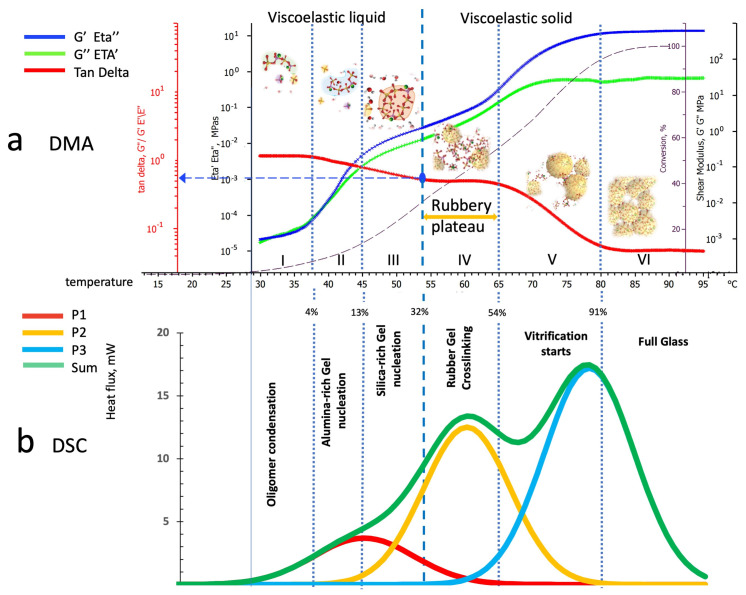
Parallel temperature scans of the alkaline-activated metakaolin paste heated at 1 °C/min: (**a**) DMA rheo-thermogram, (**b**) deconvoluted peaks from DSC calorimetric thermogram.

The semi-logarithmic diagram of the DMA rheo-thermogram of a test run at 1 °C/min has been twinned with the DSC of a specimen heated at the same rate and reported in Figure 15.

The viscoelastic rheological behavior during the polymerization of an alkaline-activated metakaolin mix with the same composition as that used in our present investigation is shown in Figure 15a. In Figure 15a, we reported the elastic and viscous components (blue and green lines) of the complex shear modulus (G′, G″) and viscosity (Eta″, Eta′), the loss tangent (tan delta, the red line) and the geopolymerization conversion percentage (the black dotted line) evaluated from the DSC calorimetric thermogram of Figure 15b.

It should be mentioned that, consistent with the dissolution rates of kaolin controlling the actual rate of formation of the oligomeric species, the loss factor has been observed to increase or stay stable during the early phases of oligomer formation to progressively decrease (increase in slurry elasticity) as polycondensation proceeds, forming alumina- and silica-rich gelled particles, reaching full gelation and final vitrification.

Figure 15a illustrates the viscoelastic transitions in the DMA rheo-thermogram associated with the progressive increase in the macromolecular complexity described in Figure 1 and sketched and paired with the behavioral zones.

The loss factor (tanδ), which is initially stable around 1.5, starts to decrease progressively when entering zone II and ending in zone III at a value of about 0.6; this denotes an increasing elastic response of the reacting slurry. Zone IV, identified as an early gelled and rubbery material, is characterized by a stable tan δ value (rubbery plateau). The rubbery plateau ends when entering zone V, which is characterized by a progressive decrease in tan δ down to 0.04, denoting a significant hardening of the material undergoing vitrification with an almost entirely elastic behavior.

From the rheological and mechanical aspects, the loss component of the complex viscosity (Eta′) increases over about three folds, starting from values of about 2.4 × 10^−5^ MPa s to 1.3 × 10^−2^ MPa s on passing from zone I to zone III just before gelation occurs. The subsequent behavior of the early gelled material is that of a soft rubber solid (storage modulus will be used to describe its hardening progression) that increasingly rises from about 1.2 MPa to 8.0 × 10^2^ MPa, passing from hard rubber to a glassy solid of zone VI.

A detailed description of the viscosities and shear moduli is given in Table 4.

By comparing the six zones with the deconvoluted peaks of Figure 15b, we notice that the first peak (P1 in red), previously associated with the linear oligomerization reactions, mainly occurs in zone I. When reactions of peak 2 (P2 in yellow) start overlapping those of P1, the rheo-thermogram of Figure 15a enters zone II, showing an abrupt increase in slurry viscosities and elastic character (decrease in tanδ), suggesting that molecular branching is occurring. The branching reactions’ concurrent contribution in forming alumina-rich gel and silica-rich gels when the progressive predominance of P2 reactions increases, leading the system to enter the third zone until complete gelation occurs at the beginning of zone IV. This zone has been previously identified as a “Rubbery plateau”, where further branching and crosslinking are supported by activating the reactions of the third peak (P3 in blue). The reactions associated with the third peak (P3) induce higher levels of crosslinking in the rubber matrix, starting the vitrification process that characterizes the fifth and sixth rheological zones of Figure 15b.

#### 4.2.2. Dynamic Mechanical Analysis and Differential Scanning Calorimetry Peaks Deconvolution in Isothermal Cure Cycles at 20 °C, 30 °C, and 40 °C

**Figure 16:**
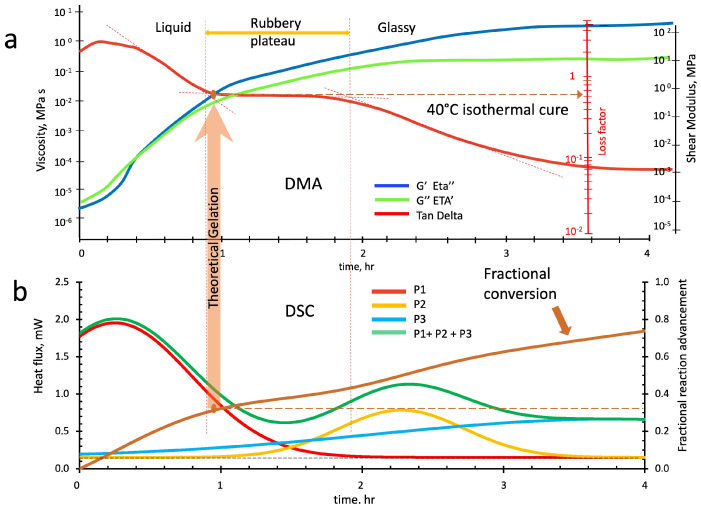
Parallel isothermal scans of the alkaline-activated metakaolin slurry at 40 °C: (**a**) DMA rheo-thermogram, (**b**) deconvoluted peaks from DSC calorimetric thermogram.

**Figure 17:**
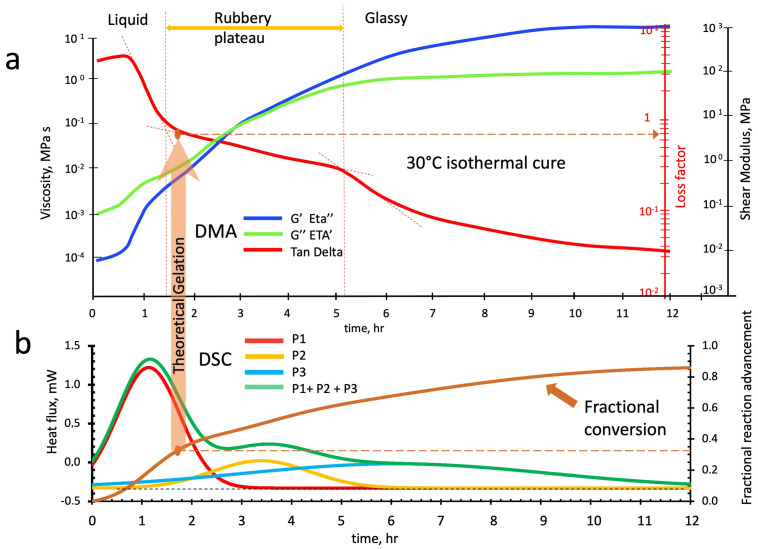
Parallel isothermal scans of the alkaline-activated metakaolin slurry at 30 °C: (**a**) DMA rheo-thermogram, (**b**) deconvoluted peaks from DSC calorimetric thermogram.

**Figure 18:**
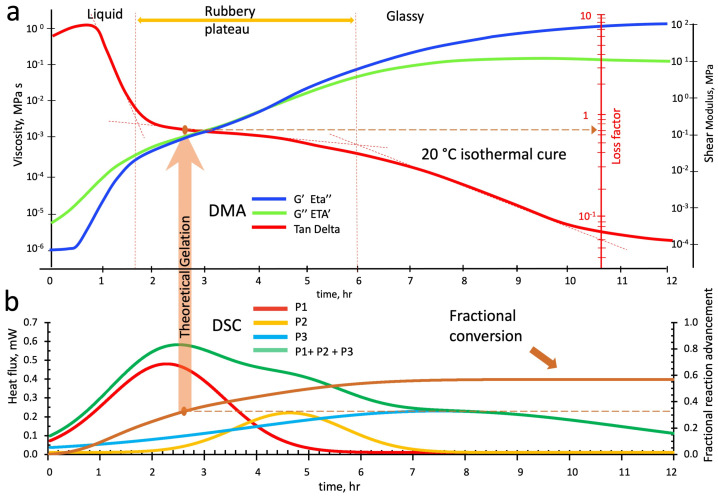
Parallel isothermal scans of the alkaline-activated metakaolin slurry at 20 °C: (**a**) DMA rheo-thermogram, (**b**) deconvoluted peaks from DSC calorimetric thermogram.

The semi-logarithmic DMA rheo-thermograms and those of the twinned DSC run at the same temperatures (40 °C, 30 °C, and 20 °C) are reported in Figure 16a,b, Figure 17a,b and Figure 18a,b, respectively. The viscoelastic analysis describes the rheological phenomena preceding gelation and vitrification. For these reasons, the loss (Eta′) and elastic (Eta″) viscosities, the elastic (G′) and loss (G″) components of the complex shear modulus, and the loss tangent (tanδ) have been monitored in twelve-hour tests.

For all tested conditions, the discussion of the relationships between the rheo-thermograms and the deconvoluted DSC peaks will be mainly focused on the first introduced behavioral zones III, IV, and V, describing the transition from viscoelastic liquid to viscoelastic solid.

Figure 16a reports the evolution of the storage (blue line) and loss (green line) viscosities (left axis in MPa s), or, equivalently, the corresponding shear moduli (right axis in MPa), and the loss tangent (red line, tan delta) as a function of the isothermal cure time at 40 °C. Although the test was conducted for twelve hours, only the first four hours are represented to better highlight the viscoelastic transitions occurring in the early stages of the fast at 40 °C geopolymerization.

In the initial stages of the curing process, the liquid slurry behaves as a dissipative viscoelastic fluid with the progression of the loss factor (red line) oscillating around a value of two to abruptly flex and rapidly decrease at hour 0.4 (see the first from left red dotted line of Figure 15a).

By comparing the viscoelastic behavioral zones of the rheo-thermogram of Figure 16a with the deconvoluted peaks of Figure 16b, we notice that the first peak (P1 in red), which has been described as associated with the linear oligomers development of zone I, mainly develops in the very early stages of the thermal cure. The reactions associated with peak 2 (in yellow) and especially those of peak 3 (in light blue) also start from the beginning of the treatment, provoking the abrupt increase in slurry viscosities and elasticity noticed at time 0.4 h, suggesting the lightweight branching and alumina-rich gel nucleation process of zone II.

The progressive predominance of P2 branching reactions’ in forming silica-rich gels increases the liquid slurry elasticity, leading the system to enter the third rheological zone preceding gelation. After that, at hour 0.9 (second red dotted line), the loss factor returns to flatten to values around 0.6–0.5, showing a plateau similar to that described for the rheo-thermogram of the dynamic temperature scan of Figure 15a. This fourth zone has been previously identified as a “Rubbery plateau”, where further branching and crosslinking are strongly supported by the reactions of the third peak (P3 in light blue). As isothermal geopolymerization proceeds, the loss factor again flexes at hour 1.9 (third red dotted line), progressively decreasing to values of 0.08–0.07. The higher level of crosslinking in the rubber matrix starts the vitrification process that characterizes the fifth and sixth rheological zones of Figure 16b.

The theoretical time to reach gelation has been evaluated from the curve of the polymerization advancement in Figure 16b at the critical conversion α_c_ = 0.333 (vertical arrow in the figure) evaluated from Equation (5), considering the presence of tetrafunctional reagent molecules in the reacting slurry. The theoretical gelation time is located at the beginning of the loss factor rubbery plateau and corresponds to the value of 0.62.

The reacting slurry loss and storage viscosities increase during the polymerization. Namely, it initially behaves as a viscoelastic liquid with loss and storage viscosities of Eta′ = 4.3 ± 0.9 × 10^−6^ MPa*s and Eta″ = 2.1 ± 0.6 × 10^−6^, respectively, which increase to values Eta′= 8.7 ± 0.5 × 10^−3^ MPa*s and Eta″ = 1.4 ± 0.4 × 10^−2^ MPa*s when reaching gelation. From this point above, the system behaves as a viscoelastic solid that initially is a lightly crosslinked rubber with storage shear modulus of G′ = 8.3 ± 1.1 × 10^−1^ MPa and a loss shear modulus of G″ = 5.0 ± 0.9 × 10^−1^ MPa that progressively harden up and become a glass with G′ = 7.9 ± 1.3 × 10^2^ MPa and G″ = 3.2 ± 0.8 × 10 MPa.

Figure 17a shows the rheo-thermogram paths for the loss and storage viscosities (Eta′ and Eta″) and shear moduli (G″ and G′) and that of the loss factor during a twelve-hour isothermal cure at 30 °C.

Even at this cure temperature, the liquid slurry behaves as a viscoelastic fluid but with a higher dissipative character than the 40 °C cure (the loss factor is 5 vs. 2). At hour 0.9, the slurry rapidly increases its elasticity, decreasing its loss factor to values of 0.8–0.9. By considering the deconvoluted peaks of Figure 17b, we notice that even at this temperature, the first peak (P1 in red) associated with the linear oligomer development mainly develops in the early stages of the geopolymerization.

Analogously with the cure at 40 °C, the reactions associated with peak 2 (in yellow) and those of peak 3 (in light blue) are concurrently present from the beginning and determine the abrupt increase in slurry viscosities and elasticity noticed to start at time 0.9 h and ending at time 1.4 h. The occurrence of light branching and nucleation of alumina-rich gel particles occurs and then, when the influence of the reactions of peak 2 becomes predominant, the system enters the incipient gelation zone. The elasticity slowly increases from hours 1.4 to 5.2, lowering the loss factor to 0.3. In this period, the loss factor path presents a “rubbery plateau” similar to those described in the previous rheo-thermograms of Figure 15a and Figure 16a. At the end of this plateau, the loss factor thermogram abruptly flexes again. It decreases, reaching a value of 0.03–0.04, indicating the beginning of the vitrification process induced by the consistent contribution of the crosslinking reactions associated with the peak 3 of Figure 17b.

The theoretical time to reach gelation is also reported in Figure 17b. The critical conversion α_c_ = 0.333 (vertical arrow in the figure) still falls at the beginning of the loss factor rubbery plateau and corresponds to the value of 0.70.

Loss and storage viscosities increase during the polymerization early stages from Eta′ = 3.5 ± 0.4×10^−4^ MPa*s and Eta″ = 8.3 ± 0.6×10^−5^ to Eta′ = 4.6 ± 0.7×10^−3^ Mpa*s and Eta″ = 6.8 ± 0.5×10^−3^ Mpa*s, respectively, when reaching the rubbery plateau. From this point above, the system is rubbery with storage shear modulus G′ = 3.4 ± 0.9×10^−1^ MPa and a loss shear modulus G″ = 2.0 ± 0.6×10^−1^ MPa that progressively with vitrification reach the final values of G′ = 9.8 ± 1.3×10^2^ MPa and G″ = 2.8 ± 0.6×10^−1^ MPa.

As reported in Figure 12 and Table 3, the fractional conversion of all the geopolymerization reactions at the end of the isothermal cure at 30 °C is about 0.86, while the partial conversions for P1, P2, and P3 are 0.35, 0.17, and 0.34, respectively.

Finally, the rheo-thermogram of Figure 18a shows the loss and storage viscosities (Eta′ and Eta″) and shear moduli (G″ and G′), and the loss factor during a twelve-hour isothermal cure at 20 °C.

The liquid slurry behaves as a viscoelastic fluid but with a very high dissipative character (the loss factor is 9 vs. 5 and 2 for the tests at 30 °C and 40 °C, respectively) that, from hours 1.0 to 1.7, rapidly decreases down to values of 0.8–0.7. By pairing this viscoelastic behavior with the deconvoluted peaks of Figure 18b, we notice that the reaction of linear oligomerization peak 1 (P1 in red) is strongly supported at this temperature by the branching and crosslinking reactions (P3 in light blue) that let the elasticity of the medium rapidly increase, entering in the zone of the incipient gelation rubbery plateau.

The occurrence of heavy branching accompanying the nucleation of alumina-rich gel particles (with Si/Al = 1) retards the formation of the silica-rich gel particles (P2 in yellow). It favors the formation of unreacted linear sialates with Si/Al = 2 or 3 (see Figure 1). The contribution of the reactions of peak 2, in fact, over the fractional advancement of the geopolymerization in the isothermal test (see Figure 12 and Table 3) is only 0.08 vs. 0.19 and 0.30 of P1 and P2, respectively. The post-cure carried out with a temperature scan up to 95 °C, conversely, indicates a massive contribution of the P2 reactions to exhaust the system residual reactivity (Figure 14a and Table 3) compared to those of the same reactions for the samples cured at 30 °C and 40 °C (Figure 14b,c): the residual fractional conversion was 0.15 vs. 0.2 and 0.2, respectively.

From hours 1.7 to 6.0, where the influence of the peak 2 branching reactions is at its maximum, the elasticity increases, lowering the loss factor to 0.5. The loss factor path presents a “rubbery plateau” even in this cure temperature. However, the theoretical time to reach gelation (2.4 h), evaluated from critical conversion α_c_ (vertical arrow in the figure), is now located in the middle of the plateau and corresponds to a loss factor of 0.70.

At the end of this plateau, the loss factor thermogram flexes again and slowly decreases, reaching a value of 0.08–0.07. This indicates the beginning of the vitrification process induced by the consistent contribution of crosslinking reactions (P3 in light blue).

Loss and storage viscosities increase during the early stages of polymerization, with a high predominance of the loss component up to the incipient gelification, namely, Eta′ = 7.2 ± 0.9 × 10^−6^ MPa*s and Eta″ = 1.1 ± 0.6 × 10^−6^, while Eta′ = 3.1 ± 0.6 × 10^−4^ MPa*s and Eta″= 2.3 ± 0.7 × 10^−4^ MPa*s when gelation occurs. From this point above, the system is rubbery with storage shear modulus G′ = 1.0 ± 0.3 × 10^−1^ MPa and a loss shear modulus G″ = 1.3 ± 0.3 × 10^−1^ MPa that progressively vitrify, reaching the final values of G′ = 1.1 ± 0.3 × 10^2^ MPa and G″ = 1.1 ± 0.6 × 10 MPa. At 20 °C and in the glassy state, the reacting species’ mobility is impaired by diffusive constraints, leaving a high fraction of unreacted material. The fractional polymerization advancement (see Figure 13 and Table 3) at this temperature is, in fact, only 0.57. According to the previous deconvolution peaks analysis, the most unreacted species could be the linear silica-rich sialates with Si/Al ratios between two and three.

## 5. Conclusions

Innovative scientific and technological approaches are needed to fully exploit the geopolymer’s chemo-physical properties and their potential use as greener materials for greener production technologies. The extrusion-based 3D printing in the construction sector operates by layer-by-layer deposition from a moving nozzle. Geopolymer materials should possess many characteristics to meet the critical parameters for 3D printing technology, such as rheological, physical, and mechanical properties. The key parameters include aluminosilicate raw materials and activator compositions, the presence of reinforcing fillers, geometrical printing variables, polymerization conditions (temperature and time), and post-processing of the 3D-printed structure.

The innovative proposed approach presented pairs DSC and DMA techniques to describe the hard setting process from raw materials oligomerization to alumina-rich/silica-rich nucleation and then rubbery and glass formation. The proposed chemorheological model is consistent with the experimental results presented, and could offer a good guidance to the 3D printing design with MKG materials.

In the early processing stages, controlling the viscosity of the extruded material is the critical parameter to be controlled. In the reacting geopolymeric paste, low-molecular-weight inorganic monomers and oligomers undergo a phase change from liquid to crosslinked glassy solid. The complexity of the concurrent chemical and rheological phenomena needs the development of mathematical models to optimize the conditions of a 3D printing process.

A Gaussian functions deconvolution method has been applied to the DSC multi-peaks thermograms to separate the kinetic contributions of the oligomer’s concurrent reactions, showing good agreement with the experimental data. The kinetic model, comprising a combination of overlapping Gaussian peaks, was associated with the initial linear oligomers formation, oligomers branching into alumina-rich and silica-rich gels, and inter- and intra-molecular crosslinking, and it has been paired with DMA viscoelastic characterization, defining an association between the thermal events and the rheology of the reacting slurry.

Gelation, associated with a dramatic viscosity increase, can start at a critical value of the degree of advancement of the geopolymerization, defining a crucial threshold in material processability. The growth and branching of the polymer chains occur in the liquid state while the system is still soluble and moldable. In contrast, the infinite network is developed after the gel point by intra-molecular reactions of the branched molecules, finally leading to an insoluble elastic crosslinked glassy solid. Vitrification prevents further reaction by reducing the mobility of the unreacted molecular species or functional groups.

The proposed kinetic and chemorheological approach for the assessment of the more idoneous processing conditions of a specific alkaline-activated aluminosilicate formulation can give insights into the characteristic viscoelastic behavioral zones of good processability, as well as the mechanical and energetical requirement for material extrusion and deposition and their final mechanical characteristics.

## Figures and Tables

**Table 1:** Metakaolin and sodium silicate solution raw materials composition [39].

Oxide ^1^	Metakaolin	Sodium Silicate
**SiO_2_**	52.30	28.36
**Al_2_O_3_**	45.20	-
**Fe_2_O_3_**	0.42	-
**K_2_O**	0.15	-
**Na_2_O**	-	8.60
**MgO**	0.04	
**H_2_O**	-	63.04

^1^ Oxides weight %.

**Table 2:** The Gaussian functions deconvolution method calculated mean values and variances of the heat released on the dynamic and isothermal tests. * One-way ANOVA at *p* < 0.05 was adopted to verify statistically significant differences in the total heat released.

DSC, Operating Conditions	Mode	ΔH Partial, J/g	ΔH Total, * J/g
Dynamic 10–95 °C scan, fresh sample	Exp.	761 ± 27	761 ± 27
	Model	760 ± 25	760 ± 25
Iso 20 °C	Exp.	511 ± 32	777 ± 19
	Model	524 ± 28	800 ± 38
Iso 30 °C	Exp.	726 ± 24	762 ± 26
	Model	712 ± 15	747 ± 31
Iso 40 °C	Exp.	748 ± 25	759 ± 25
	Model	751 ± 17	763 ± 35
Dynamic 10–95 °C scan After 20 °C cure	Exp.	266 ± 12	-
	Model	276 ± 27	-
Dynamic 10–95 °C scan After 30 °C cure	Exp.	36 ± 6	-
	Model	35 ± 9	-
Dynamic 10–95 °C scan After 40 °C cure	Exp.	11 ± 3	-
	Model	12 ± 2	-

**Table 3:** Mean values and variances of the reaction’s fractional advancements for the dynamic and isothermal tests were calculated using the Gaussian functions deconvolution method.

DSC, Operating Conditions	P1 Fractional Conversions	Fractions Sum	P2 Fractional Conversions	Fractions Sum	P3 Fractional Conversions	Fractions Sum
Dynamic 10–95 °C scan on fresh sample	0.12 ± 0.04	0.12 ± 0.04	0.36 ± 0.04	0.36 ± 0.04	0.52 ± 0.07	0.52 ± 0.13
Iso 20 °C+ Dynamic 10–95 °C	0.19 ± 0.04	0.22 ± 0.03	0.08 ± 0.02	0.23 ± 0.09	0.30 ± 0.07	0.51 ± 0.12
	0.03 ± 0.01		0.15 ± 0.04		0.21 ± 0.03	
Iso 30 °C+ Dynamic 10–95 °C	0.35 ± 0.05	0.39 ± 0.05	0.17 ± 0.08	0.19 ± 0.02	0.34 ± 0.09	0.42 ± 0.09
	0.00 ± 0.00		0.02 ± 0.01		0.04 ± 0.01	
Iso 40 °C+ Dynamic 10–95 °C	0.36 ± 0.00	0.36 ± 0.06	0.15 ± 0.04	0.17 ± 0.05	0.46 ± 0.10	0.47 ± 0.10
	0.00 ± 0.00		0.02 ± 0.01		0.01 ± 0.01	

**Table 4:** Viscosities, shear moduli, and loss factors identifying the six chemorheological behavioral zones of the reacting slurry heated at 1 °C/min.

ZONE	Loss Viscosity, MPa*s	Storage Modulus, MPa	Loss Factor, tanδ
I	2.4 ± 0.4 × 10^−5^	-	1.1 ± 0.1
	7.7 ± 0.3 × 10^−5^		1.3 ± 0.3
II	-	-	-
	2.3 ± 0.6 × 10^−3^		0.75 ± 0.05
III	-	-	-
	1.3 ± 0.5 × 10^−2^		0.51 ± 0.07
IV	-	-	-
		1.2 ± 0.5 × 10	0.43 ± 0.04
V	-	-	-
		6.6 ± 0.6 × 10^2^	0.05 ± 0.01
VI	-	-	-
		8.2 ± 0.6 × 10^2^	0.04 ± 0.01

## Data Availability

Data are contained within the article.

## Supplementary Materials

The following supporting information can be downloaded at: https://www.mdpi.com/article/10.3390/polym16020211/s1.
