# Supplementary material for: Thermokinetic and Chemorheology of the Geopolymerization of an Alumina-Rich Alkaline-Activated Metakaolin in Isothermal and Dynamic Thermal Scans
Source: Polymers (Basel). 2024 Jan 11;16(2):211. doi: 10.3390/polym16020211 (PMC10819406; doi:10.3390/polym16020211)

Additional materials (from ref.39)

Geopolymer raw material composition

Materials (wt%)	Geopolymer
Metakaolin	37.5
NaOH	7.2
Sodium silicate	55.3

X-ray Diffraction Characterization

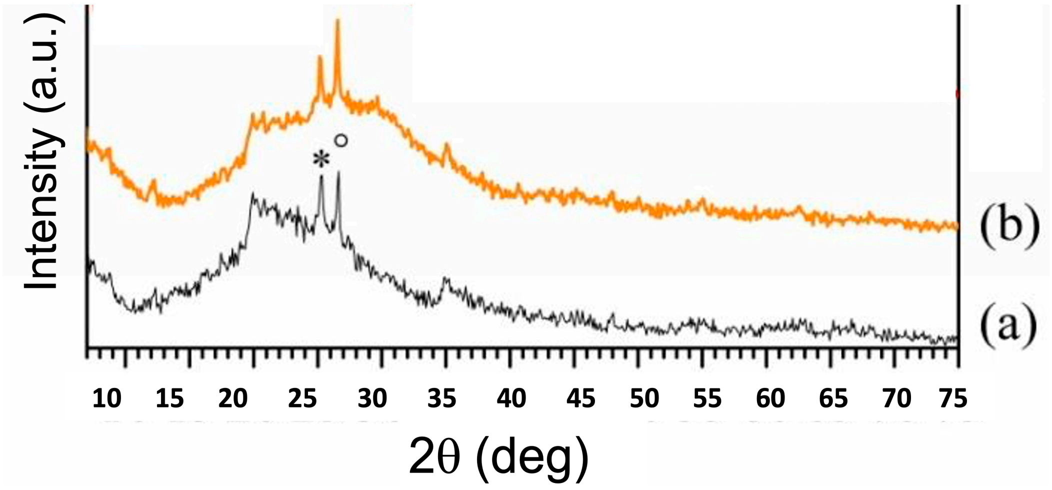

X-ray powder diffraction patterns of (a) metakaolin; (b) Geopolymer. Crystalline phases: * = anatase; ° = quartz (see Table 1 of metakaolin composition).

The diffraction patterns of the metakaolin specimen (a) used for the preparation of the geopolymer sample (b) reveal their predominantly amorphous character, evident in a large halo centred at 2θ ≈ 20–25° and by some minor diffraction peaks revealing the presence of residual kaolinite (2θ ≈ 19–21°). Small impurities are probably represented by TiO2 (anatase) and SiO2 (quartz), whose crystalline phases can be identified by the sharp diffraction peaks at 25.3° and 27.5°, respectively.

EDS spectrum

Element Symbol	Atomic Conc.	Weight Conc.	Oxide Symbol	Stoich. wt Conc.
O	58.95	45.92		
Si	24.38	33.35	SiO_2	67.00
Al	8.30	10.91	Al_2O_3	19.23
Na	8.28	10.89	Na_2O	19.22

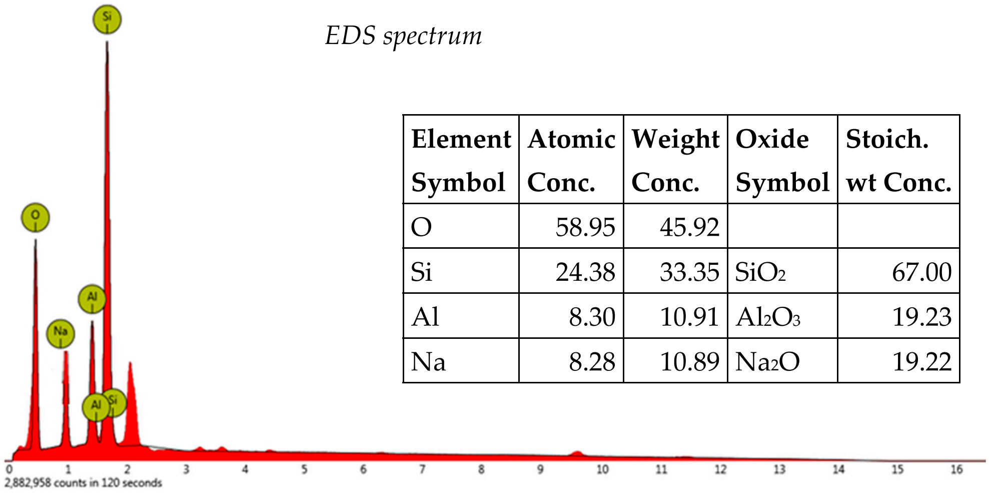

Supplement: Supplementary file 1 [file polymers-16-00211-s001.zip › polymers-2801246-supplementary.pdf]
